# General self-efficacy and diabetes management self-efficacy of diabetic patients referred to diabetes clinic of Aq Qala, North of Iran

**DOI:** 10.1186/s40200-016-0285-z

**Published:** 2017-02-15

**Authors:** Hajar Dehghan, Abdurrahman Charkazi, Ghorban Mohammad Kouchaki, Bagher Pahlevan Zadeh, Bibi Azizieh Dehghan, Mohammad Matlabi, Morteza Mansourian, Mostafa Qorbani, Omid Safari, Tahereh Pashaei, Babak Rastegari Mehr

**Affiliations:** 1 Laboratory Sciences Research Center, Golestan University of Medical Sciences, Gorgan, Iran; 2 Environmental Health Research Center, Golestan University of Medical Sciences, Gorgan, Iran; 3 Department of Biostatistics, School of Allied Medical Sciences, ShahidBeheshti University of Medical Sciences, Tehran, Iran; 4 AleJalil Hospital, AqQala Health Center, Golestan University of Medical Sciences, AqQala, Iran; 5 Public Health Department, School of Health, Gonabad University of Medical Sciences, Gonabad, Iran; 6 Health Management and Economics Research Center, Iran University of Medical Sciences, Tehran, Iran; 7 Department of Health Education and Promotion, Iran University of Medical Sciences, Tehran, Iran; 8 Non-communicable Diseases Research Center, Alborz University of Medical Sciences, Karaj, Iran; 9 Chronic Diseases Research Center, Endocrinology and Metabolism Population Sciences Institute, Tehran University of Medical Sciences, Tehran, Iran; 10 Departments of Pediatrics, School of Medicine, Alborz University of Medical Sciences, Karaj, Iran; 11 Social Determinants of Health Research Center and Public Health Department, Kurdistan University of Medical Sciences, Sanandaj, Iran; 12 Abadan School of Medical Sciences, Abadan, Iran

**Keywords:** Diabetes, Diabetes self-efficacy, Glycemic control, Aq Qala

## Abstract

**Background:**

Self-efficacy is one of the factors involved in successful self-care of diabetic patients. The aim of this study was to evaluate general self-efficacy and diabetes management self-efficacy and to determine their association with glycemic control in diabetic individuals, referred to the diabetes clinic of Aq Qala city, North of Iran.

**Methods:**

In this cross-sectional study, 251 type 2 diabetes mellitus patients were enrolled using census method. Data collection tools consisted of Sherer General Self-Efficacy Scale (SGSES) and Diabetes Management Self-Efficacy Scale (DMSES) with minor demographic adjustments and hemoglobin A_1_C test. Data were analyzed using descriptive statistics and analytical techniques include independent *t*-test, Spearman correlation coefficient and linear regression were applied for further data analysis.

**Results:**

The mean and standard deviation age of subjects was 56.17 ± 10.45 years. The mean level of HbA_1_C of studied subject was 8.35 ± 2.02%. There was a negative correlation between age and general self-efficacy and diabetes self-efficacy while, there was a positive correlation between general self-efficacy and diabetes self-efficacy (*P* < 0.001). Results of the regression analysis showed that duration of the disease was the only variable which had a significant effect on the level of hemoglobin A1C (*P* < 0.001), so that for each year of having the disease, the level of hemoglobin A_1_C increased by 0.084% (CI 95% = 0.048–0.121).

**Conclusions:**

General self-efficacy and diabetes self-efficacy does not affect glycemic control in diabetic individuals. The duration of the disease is the only affecting variable on glycemic control by its worsening in diabetic individuals. Interventions are recommended to help glycemic control in individuals who are having this disease for longer periods. Moreover, further studies on the affecting factors on poor glycemic control of diabetic patients as well as the role of time variable, are recommended.

## Background

Diabetes is the fifth leading cause of death In the Western societies and the fourth most common cause of physicians’ visit [1]. Prevalence of diabetes and impaired glucose is increasing in Iran. Its prevalence among 25 to 70 years old was 11.4% (4.5 million people) in 2011 [2]. Diabetes mellitus is a chronic disease that can affect all aspects of life. Much of the care plan for this disease is interwoven with the daily life behaviors, thus diabetic individuals are the most responsible for control and management of the disease [3]. Self-efficacy is one of the contributing factors which can have a major role in the success of diabetic control and self-care. The researchers believe that self-efficacy is an appropriate framework to understand and predict the behavior and commitments of patients to self-care in the treatment of diabetes [4, 5].

Self-Efficacy (SE) was first proposed by the psychologist, Albert Bandura. According to Bandura, self-efficacy is a belief of individuals in their abilities to carry out a successful practice and is a theory in itself, as well as a structure of the social cognitive theory. The self-efficacy theory argues that people will take action when they believe they are able to do it and will avoid and action when they believe they may fail. Self-efficacy is the prerequisite of a behavior and should be considered as an independent part of basic skills [6]. In total, Bandura believes that self-efficacy is the main structure in predicting individuals’ behavior change and usually the ones that show a high level of behavioral changes have higher efficacy [7]. Self-Efficacy(SE) has prominent role in diabetes self – management and predicts its outcome. In their study Didarloo et al. reported that self-efficacy explained 11.4% of variance regarding to diabetes self care and 31.3% variance of diabetes self care behavioral intention [8]. Diabetes management self-efficacy (DMSE) which developed by Kara and colleagues measures the diabetic patients confidence regarding to diet, exercise and medical treatment [9]. Luszczynska et al. study on 8796 subjects from five countries revealed that General Self-Efficacy (GSE) has notable association with specific self-efficacy; optimism, self-regulation, and self-esteem; whereas the converse associations with depression and anxiety [10].

The results of Shahab - Jahanlou and Alishan- Karami study showed that self-efficacy has a strong relationship with quality of life in diabetic patients [11]. O’Hea and colleagues demonstrated that the eating behaviors of diabetic patients are associated with their self-efficacy [12]. The study results of Alato also showed that self-efficacy is the strongest predicting factor in determining the patients’ glucose level [13].

The results of the literature review has shown the effect of diabetes mellitus self-efficacy in glycemic control, but no study was found on the role of general self-efficacy (GSE) and diabetes management self-efficacy (DMSE) and their association with glycosylated hemoglobin. Since, ethnicity and cultural factors can affect the self-efficacy and the vast majority of diabetic patients in the city of Aq Qala are Turkmen.

### Objectives

This study aimed to determine the GSE and DMSE and they relationship with glycemic control in diabetic patients, referred to the diabetes clinic of Aq Qala, Iran.

## Methods

This was a cross-sectional, descriptive-analytical study which conducted between September to December 2014 in Aq Qala city, North of Iran. The study population consisted of all patients with a positive type II diabetes mellitus diagnosis, visiting the Aq Qala diabetes clinic with active health records. The patients were selected by census and finally, among 290 patients with active health records at the clinic, 251 patients were enrolled and 39 patients were withdrawn from the study.

Inclusion criteria included having an active clinical record at the diabetes clinic of Aq Qala, the ability to communicate verbally, non-hospitalization during the interview, having Turkmen ethnicity, at least 1 year elapsed time from the diagnosis and no history of mental retardation or other psychological disorders such as mood and anxiety disorders before the diagnosis of diabetes or severe psychological disorder after the diagnosis of diabetes.

Data collection was conducted using the following questionnaire: demographic information questionnaire, Sherer et al. general self-efficacy scale (GSES) [14] and diabetes management self-efficacy scale (DMSES) [6]. Demographic and clinical data included subjects’ age, gender, education level, occupation, marital status, number of children and duration of the disease. Sherer et al. GSES is consisted of 17 statements. Scoring of this scale was based on a 5 point index which was rated as follows: Strongly Disagree: 1, against: 2, median: 3, agree: 4, strongly agree: 5 points, so that the highest and lowest points in this questionnaire were 85 and 17, respectively. The validity and reliability of the questionnaire was approved in our country by Asgharnejad et al. [15].

DMSES contains 19 questions to measure the patient’s ability to manage their disease. Each person determines his/her capabilities on each question by drawing a circle around one of the numbers between 0 (completely unable) to 10 (completely able) and each answer was then rated from zero to 10. The Reliability and validity of this study in our country has been approved by Haghayegh and Noorozi [15, 16]. Measures of the current study by two Turkmen researchers translated to Turkmen language from Persian. After that, the measures were back translated independently by three bilingual Turkmen literacy specialists to Persian. After some adjustment on the questionnaires, the 12 subjects with type 2 diabetes referring to the diabetes clinic (non-participants) were given the questionnaire and completed it two times within two weeks. Cronbach’s Alpha was used to evaluate the reliability of both questionnaires and both questionnaires were confirmed with the correlation coefficient of α = 0.996 and α = 0.978 for Sherer general self-efficacy questionnaire and diabetes management self-efficacy, respectively.

After approval of the university’s deputy of Research and Technology and the authorization of Aq Qala diabetes clinic located in the Ale Jalil hospital, the questionnaires were completed by two native nursing MSc and BSc graduate. In this regard, first, the objectives of the study were presented to the patients and after obtaining oral and written consents, they were asked to participate in the study. The questionnaire was completed in the presence of the questioner and then 2 ml of venous blood samples from each patient was prepared and sent to the Aq Qala health center laboratory for HbA1c analysis.

### Statistical analysis

The obtained data were analyzed using SPSS version 18 for central indices (mean) and dispersion (standard deviation). Pearson correlation test was used to determine the relationship between GSE and DMSE. Also, the linear regression model was used to assess the association of GSE and DMSE with HbA_1_c levels. The backward method was used for regression analysis. To determine the relationship between GSE and DMSE with gender variable, independent *t*-test was used and Spearman correlation coefficient rank test was applied to examine the relationship between GSE and DMSE with variables of age and duration of the disease.

One way variance analysis was applied to evaluate the association between GSE and DMSE with variables of occupation and level of education. Mann-Whitney was applied In order to examine the relationship between GSE and DMSE with A1C levels. Shapiro-Wilk test was applied for checking whether a continuous variable is normally distributed. A1c levels divided into groups; Good control 7.5% and below and poor level which was more than 7.5%. *P*-value of less than 0.05 was considered as statistical significance in all tests.

## Results

Among all the study cases, 195 patients (77.7%) were male and 249 (99.2%) were married. The subjects were aged 25–85 with mean (SD) of 56.17 ± 10.45 years. Duration of diabetes was 8.66 ± 6.68 years and the mean of HbA_1_C in subjects was found as 8.35 ± 2.02%. Also, 168 patients were (66.9%) uneducated, 112 patients (44.6%) had rural insurance, 129 (51.4%) had retinopathy and 185 subjects (73.7%) were taking medication for the diabetes control (Table 1).

**Table 1:** Demographic profile of the participants

Variables		Number of individuals	Percentage
Education Level	Uneducated	168	66.9
	Primary school	51	20.3
	Guidance school	6	2.4
	High School	20	8
	Bachelor Degree	6	2.4
Type of Insurance	Rural	112	44.6
	Treatment services	75	29.9
	Social Security	48	19.1
	Armed Forces	13	5.2
	Others	3	1.2
Complications	Retinopathy	129	51.4
	Neuropathy	47	18.8
	Nephropathy	3	1.2
	Combination of Complications	4	1.6
	No complication	68	27.1
Type of Medication	Pill	185	73.7
	Insulin	33	13.1
	Insulin and Pill	15	6
	None	18	7.2
HbA1c level	Good	82	32.8
	Poor	169	67.2

The mean and Standard deviation of GSE and DMSE in the subjects were 2.64 ± 1.08 (of 5) and 5.49 ± 1.99 (of 10), respectively. Mann-Whitney test results showed that although the mean and standard deviation of GSE and DMSE in the group with good HbA_1_C level was more than the group with poor HbA_1_C control level, these differences were not statistically significant (Table 2).

**Table 2:** The Mean and standard deviation of GSE and DMSE in patients with good and poor glycemic control

Variables	Good HbA1c control	Poor HbA1c control	*P*-value
	Mean and SD	Mean and SD	
GSE	2.77 ± 1.10	2.57 ± 1.06	0.165
DMSE	5.68 ± 2.03	5.39 ± 1.97	0.233

Spearman’s rank correlation coefficient results showed a negative correlation between age and GSE and DMSE as well as a positive correlation between GSE and DMSE (Table 3).

**Table 3:** Spearman correlation coefficient test results, HbA1C hemoglobin level, GSE and DMSE

	Age	HbA_1_c	GSE	DMSE
Age	-			
HbA_1_c	−0.890^a^	-		
GSE	−0.379^a^	−0.065	-	
DMSE	−0.239^a^	−0.07	0.509^a^	-

^a^significant at level 0.01

Hemoglobin A_1_C level was in the range of about 3 to 13.4% with mean (SD) of 8.35 ± 2.02%, where the Shapiro-Wilk test showed its normal distribution. Linear regression model was used to assess of the relationship between hemoglobin A1C with factors such as gender, general self-efficacy, diabetes mellitus self-efficacy and the duration of diabetes. The results showed that the years of dealing with diabetes had a significant effect on hemoglobin A1C levels (*P* < 0.001), so that for each year of having diabetes, the patient’s hemoglobin A_1_C level was increased by 0.084% (CI 95% = 0.048–0.121) (Table 4).

**Table 4:** Results of the linear regression

Predictor	*b* (95% CI)	SE B	β	*p* value
Intercept	7.652 (7.227–8.023)	0.202	0.279	<0.0001
Duration of Diabetes	0.084 (0.048–0.121)	0.018		<0.0001

Hemoglobin A_1_C = 7.652 (0.084× Duration of Diabetes)

## Discussion

The results showed a high correlation between general self-efficacy scale and diabetes self-efficacy scale, but there was no relationship between these two scales and glycemic control (Glycosylated hemoglobin). This non-compliance may be due to the direct influence of glycemic control in patients’ self-care, while the general self-efficacy and diabetes self-efficacy cannot have a direct effect on glycemic control [17]. It is recommend that when glycemic control is assessed as an outcome variable in clinical and research objectives, the adequacy of the treatment regimen and drug consumption of patients should be considered, as well as evaluating the treatment and medication adequacy in each examination, the lack of glycemic control and unsatisfactory results should be highlighted [17]. This study did not evaluate patients’ self-care which was a limitation for this study.

The results also showed a significant negative correlation between the duration of diabetes and diabetes self-efficacy. This means that people with a long history of diabetes had lower diabetes self-efficacy. This could be due to the fact that as time passes, patients become more exhausted about their disease, therefore their self-efficacy will also decrease. According to Bandura, the failure experience is one of the self-efficacy theory constructs, thus its decrease results in lower possibility of increasing the self-efficacy. In Chih et al. study, there was a negative correlation between diabetes perceived self-control scale and duration of type 1 diabetes mellitus [18] which is consistent with our study.

Moreover, there was a significant positive correlation observed between the duration of diabetes and glycosylated hemoglobin levels which shows that as the duration of disease increases, blood sugar control becomes worse. The results of Paula et al. study in the United States showed that poor glycemic control is accompanied with long duration of diabetes. They believed that failure of patients in achieving the optimum level of glycosylated hemoglobin over time, leads to frustration, disappointment and thus may reduce their self-efficacy [19].

The results of linear regression analysis showed that only duration of diabetes variable was affecting the level of glycosylated hemoglobin in this study. This means that after a year from developing diabetes, the hemoglobin levels increased 0.084%. In other words, when two patients had 1 year difference of having diabetes, with the same condition their mean levels of hemoglobin A_1_C was different by 0.084%.In the other hand, duration of diabetes worsens patients’ glycemic control which is consistent with findings of Trief et al. study [19].

## Conclusion

The results showed that general self-efficacy and diabetes self-efficacy do not affect glycemic control of patients while duration of diabetes development is the only variable that affects glycemic control by worsening this variable in diabetic patients. Based on Green and Kreuter health promotion model, PRECEDE, predisposing, reinforcing and enabling factors are most important issues in any healthy behaviors [20, 21]. Predisposing factors include the people belief, knowledge and attitude regarding to the disease which are necessary for healthy actions. Self-efficacy is considered as a predisposing factor that can be deteriorated in chronic diseases like Diabetes. Incresaing in self-confidence levels of diabetic patients can set the stage for glycemic control. Interventions are recommended for glycemic control of patients with longer duration of disease. In their study, Borhani et al. found that predisposing, reinforcing and enabling factors increased the self-care behaviors in diabetes patients [22, 23]. Moreover, further studies are recommended on the factors of glycemic control deterioration in type 2 diabetic patients and time variable.

### Study limitations and strengths

In spite of the methodological strengths of the current study such as biochemical verification using hemoglobin A1C and census sampling, there are some limitations needed to be acknowledged in generalize of the current study. First, the cross-sectional design of the study. Second, all of them were measured by self-report that has its natural limitation.

## Abbreviations

DMSES: Diabetes Management Self-Efficacy Scale (DMSES)
SGSES: General Self-Efficacy Scale.
